# Patients’ Acceptance and Intentions on Using Artificial Intelligence in Dental Diagnosis: Insights From Unified Theory of Acceptance and Use of Technology 2 Model

**DOI:** 10.1016/j.identj.2025.103893

**Published:** 2025-09-06

**Authors:** Sachin Naik, Sajith Vellappally, Mohammed Alateek, Yasser Fahad Alrayyes, Abdul Aziz Abdullah Al Kheraif, Talal Mughaileth Alnassar, Ziyad Mohammed Alsultan, Nebu George Thomas, Avneesh Chopra

**Affiliations:** a Dental Biomaterials Research Chair, Dental Health Department, College of Applied Medical Sciences, King Saud University, Riyadh, Saudi Arabia; b Dental Health Department, College of Applied Medical Sciences, King Saud University, Riyadh, Saudi Arabia; c Dental University Hospital, King Saud University, Medical City, Riyadh, Saudi Arabia; d Department of Prosthetic Dental Sciences, College of Dentistry, King Saud University, Riyadh 11545, Saudi Arabia; e Department of Periodontology, Pushpagiri College of Dental Sciences, Thiruvalla, Kerala, India; f Department of Periodontology, Oral Medicine and Oral Surgery, Institute for Dental and Craniofacial Sciences, Charité–University Medicine Berlin, Corporate member of Freie Universität Berlin, Humboldt–Universität zu Berlin, and Berlin Institute of Health, Berlin, Germany; g Department of Conservative Dentistry and Periodontology, Medizinische Hochschule Brandenburg (MHB) Theodor Fontane, Brandenburg an der Havel, Germany

**Keywords:** Artificial intelligence, Dental diagnosis, UTAUT2, Structural equation modelling, Healthcare technology, Behavioural intention

## Abstract

**Introduction and aims:**

Artificial intelligence (AI) is transforming dental care by enhancing diagnostic accuracy, efficiency, and patient experience. This study aimed to assess dental patients’ acceptance, perceptions, and concerns regarding AI-powered diagnosis using the Unified Theory of Acceptance and Use of Technology 2 (UTAUT2) framework through structural equation modelling (SEM).

**Methods:**

A cross-sectional study was conducted among dental patients at King Saud University Dental Hospital, Riyadh. Data were collected using a structured questionnaire based on the UTAUT2 framework. Confirmatory factor analysis was performed to assess the validity and reliability of the measurement model. SEM was then utilized to evaluate the relationships between UTAUT2 constructs and patients’ behavioural intention toward AI-powered dental diagnosis.

**Results:**

A total of 505 participants responded to the questionnaire. SEM indicated a strong model fit (GFI = 0.900, Comparative Fit Index = 0.906, Tucker–Lewis Index = 0.907, root mean square error of approximation = 0.083). Performance expectancy (*β* = 0.32, *P* < .001), social influence (*β* = 0.56, *P* < .001), and facilitating conditions (*β* = 0.34, *P* < .001) significantly predicted behavioural intention. The model explained 68.1% of the variance (*R*² = 0.681), highlighting key drivers of AI adoption. While 60% of participants trusted AI dental diagnosis, 55% expressed concerns about data privacy and accuracy.

**Conclusion:**

This study demonstrates that performance expectancy, social influence, and facilitating conditions are key factors influencing the acceptance of AI in dental diagnosis. Addressing these factors can help develop user-friendly AI tools in dentistry, thereby improving adoption. Future research should focus on clear communication about AI and patient education to alleviate concerns and promote equitable and widespread use.

**Clinical relevance:**

Acceptance of AI in dentistry depends on performance, motivation, and trust. Understanding these factors can guide the user-centred development of AI technologies, enhancing their integration into diagnostics and improving patient outcomes.

## Introduction

Integrating artificial intelligence (AI) in dentistry has emerged as a transformative force, reshaping diagnostic practices and patient care. Globally, AI is increasingly used to improve diagnostic accuracy in dentistry, such as accurately analysing radiographs for caries and periodontal disease.^1^ This capability is particularly beneficial as it reduces the likelihood of human error, thereby improving patient outcomes. AI systems can analyse vast amounts of patient data and imaging to identify conditions such as dental caries and periodontal disease with remarkable accuracy, often surpassing traditional diagnostic methods.^2^^,^^3^ AI’s personalized treatment recommendations enhance patient-centred care.^3^

In Saudi Arabia, the adoption of AI in healthcare, including dentistry, is gaining momentum. The Saudi government is actively promoting the use of technology to enhance healthcare services, particularly in response to challenges such as an ageing population and the increasing prevalence of chronic diseases.^4^

Despite the promising applications of AI in dentistry, significant concerns remain regarding its implementation. AI in healthcare raises important privacy, security, and ethical issues.^5^ Patients may have concerns about the accuracy of AI-powered diagnosis and the potential for technology to replace human judgment in clinical settings. These worries highlight the importance of assessing patient perceptions and acceptance of AI technologies, as understanding these factors is crucial for their successful integration into dental practice.^6^

Significant research gaps exist in understanding patient perceptions and acceptance of AI in dental settings, which differ from general healthcare due to unique interpersonal dynamics.^7^ While the technical aspects of AI are well studied, human factors such as how dental professionals communicate AI’s benefits and limitations to patients remain underexplored.^8^ Ethical concerns, particularly regarding patient privacy and data security in dental AI applications, also lack targeted investigation despite insights from broader healthcare research.^9^ Demographic factors (eg, age, education, cultural background) influencing AI acceptance are rarely disaggregated in existing studies.^10^ The application of theoretical frameworks such as the Unified Theory of Acceptance and Use of Technology 2 (UTAUT2) to assess AI acceptance in dentistry is limited, hindering actionable insights into factors like performance expectancy (PE) and social influence (SI). Addressing these gaps is crucial to fostering trust, tailoring AI integration, and developing ethical guidelines for dentistry.

While AI has the potential to revolutionize diagnostic practices and improve patient outcomes, understanding patient acceptance, perceptions, and concerns is crucial for successful implementation. Using the UTAUT2 framework to assess these factors can provide valuable insights into effectively integrating AI technologies into dental practice, ultimately enhancing the quality of care delivered to patients.^11^ Hence, the present study aimed to assess dental patients’ acceptance, perceptions, and concerns toward AI-powered diagnosis using the UTAUT2 framework and structural equation modelling (SEM).

### Study the hypothesis and theoretical framework

The most comprehensive model for predicting information technology acceptance was UTAUT,^12^ until UTAUT2 emerged. UTAUT2’s improved explained variance makes it ideal for this study.^11^^,^^13^ The seven-construct UTAUT2 scale assesses PE, effort expectancy (EE), SI, FC, hedonic motivation (HM), price value, and habit (HT). It includes two endogenous variables: Behavioural intention (BI) and use behaviour.

Recent studies in digital health and AI adoption have demonstrated the applicability of the UTAUT2 model in evaluating emerging technologies. PE and HM have been identified as strong predictors of acceptance of digital health tools.^14^ SI and EE have shown significant effects on BI to adopt AI-assisted clinical decision systems.^15^ These findings emphasize the importance of considering various behavioural determinants when assessing the integration of AI technologies in patient-centred healthcare.

Based on this theoretical foundation, the following hypotheses (**H**) were developed (Supplementary Table 1):

• **H1:** PE will have a positive impact on BI to adopt AI-powered dental diagnosis.
• **H2:** EE will have a positive impact on BI to adopt AI-powered dental diagnosis.
• **H3:** SI will have a positive impact on BI to adopt AI-powered dental diagnosis.
• **H4:** FC will have a positive impact on BI to adopt AI-powered dental diagnosis.
• **H5:** HM will have a positive impact on BI to adopt AI-powered dental diagnosis.
• **H6:** HT will have a positive impact on BI to adopt AI-powered dental diagnosis.

BI reflects the extent to which an individual is willing to engage in a specific action, embracing the shift from specialized scanning to a combination of AI assistance and novice operation. It serves as the primary dependent variable for the hypotheses outlined above.

## Materials and methods

**Study design and participants.** This cross-sectional questionnaire study was conducted at King Saud University Dental Hospital, Riyadh, Saudi Arabia, between December 2024 and March 2025. Eligible participants were adult patients (≥18 years old) actively undergoing dental treatment at the hospital, including restorative, prosthodontic, endodontic, periodontal, or orthodontic procedures. Individuals receiving one-time emergency treatments (eg, extractions without follow-up) or those with cognitive or psychiatric conditions impairing informed consent were excluded.

**Participant recruitment.** Participant recruitment followed a standardized protocol. Potential participants were systematically identified through the hospital’s patient management system and invited to participate via secure digital channels (email or WhatsApp) during their treatment period. The electronic questionnaire, hosted on Microsoft Forms, was distributed to 1,000 consecutively identified eligible patients over the study period, yielding 505 complete responses. To ensure data completeness, the survey platform required responses to all items, preventing partial submissions.

The survey followed Dillman’s Tailored Design Method, using five strategic contact points: (1) a prenotification email, (2) a formal invitation with a questionnaire link, (3) a thank-you/reminder email after 1 week, (4) a 2-week follow-up with the questionnaire link, and (5) a final reminder via WhatsApp at 3 weeks. All communications featured personalized salutations, institutional branding, and researcher contact information, resulting in a 50.5% response rate (505/1000) while ensuring data completeness through mandatory responses.

**Sample size calculation.** An initial power analysis using G*Power (*α* = 0.05, power = 80%, *f*² = 0.15) indicated a minimum of 129 participants for multiple regression analysis. For the SEM of our 32-parameter UTAUT2 model, we adopted a conservative observation-to-parameter ratio of 15:1, resulting in a minimum requirement of 480 participants to ensure stable and reliable estimates. To enhance the robustness of our analysis and account for potential data exclusions, we aimed for a slightly larger sample and ultimately obtained 505 valid responses, exceeding the recommended threshold. This sample size was further supported by pilot testing (*n* = 30), which showed strong internal consistency (Cronbach’s *α* > 0.80) and dental-specific effect sizes consistent with previous studies on healthcare technology adoption.

**Measures.** The survey was based on the UTAUT2 framework,^13^ adapted for dental healthcare contexts. The original English questionnaire underwent a rigorous translation process: (1) bilingual experts performed the Arabic translation; (2) independent back-translation verified conceptual equivalence; and (3) a reconciliation committee resolved discrepancies. Pilot testing with 30 dental patients confirmed cultural appropriateness, prompting terminology refinements (eg, ‘mobile apps’ → ‘dental health technologies’). All constructs used 7-point Likert scales (1 = strongly disagree to 7 = strongly agree). The final instrument demonstrated excellent reliability in our sample, with Cronbach’s *α* ranging from 0.82 to 0.91 across all UTAUT2 constructs.

**Data analysis.** Data analysis was performed using SPSS 28.0 for descriptive and reliability analyses, and AMOS 24.0 for advanced modelling.

**Confirmatory factor analysis (CFA).** CFA was performed to assess the model fit of the latent variable BI. The following fit indices were examined: Chi-square minimum discrepancy function divided by degrees of freedom (CMIN/DF), Chi-square (*χ*²), Comparative Fit Index (CFI), Tucker–Lewis Index (TLI), and root mean square error of approximation (RMSEA). An RMSEA value below 0.05 indicated a good fit, while CFI and TLI values above 0.90 were considered acceptable.^16^ Composite reliability (CR) was used to assess internal consistency, with values ideally exceeding 0.7. Convergent validity was established when all standardized item loadings exceeded 0.70 and the average variance extracted (AVE) for each construct surpassed the recommended threshold of 0.5, as suggested by Fornell and Larcker.^17^

**SEM.** SEM was employed to validate the UTAUT2 framework by assessing the relationships between latent constructs and the endogenous variable BI. Maximum likelihood estimation with 2,000 bootstrap samples was used to generate stable parameter estimates, identifying significant pathways (*P* < .01).

**Fig. 1:**
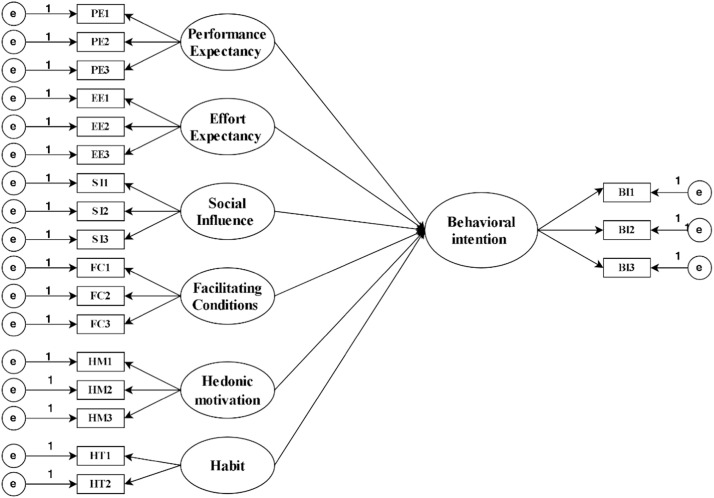
AMOS model diagram for the UTAU2 framework.

Figure 1 outlines the measurement model structure, categorizing latent constructs and their respective indicators (eg, PE1-PE3 for PE, B1-B3 for BI). This framework aligns with UTAUT2 theory, illustrating the hypothesized relationships between technology adoption drivers and behavioural outcomes.

## Results

The study surveyed 505 participants, the majority of whom were Saudi nationals (84%) and predominantly male (56%). The age distribution skewed toward younger adults, with 47% aged 18 to 30 and 36% aged 31 to 45; older adults (>60 years) comprised only 3% of the sample. Education levels were relatively high, with 48% holding professional degrees and 31% possessing graduate qualifications. Approximately 57% of respondents reported prior experience with AI technologies, indicating an overall sample with above-average technological familiarity (Table 1).

**Table 1:** Demographic characteristics of participants (*n* = 505).

Variable	Category	Frequency (*n*)	Percentage (%)
Age (y)	18-30	236	47
	31-45	182	36
	46-60	71	14
	>60	16	3
Gender	Male	284	56
	Female	221	44
Nationality	Saudi	426	84
	Non-Saudi	79	16
Education level	High school	45	9
	Diploma	61	12
	Graduate	159	31
	Professional	240	48
Prior use of AI technologies in healthcare or other sectors	Yes	286	57
	No	219	43

Participants perceived AI-powered dental diagnosis favourably, with PE scoring highest (mean = 3.41-3.73), indicating a strong belief in AI’s clinical benefits. EE (mean = 3.43-3.51) and HM (mean = 3.33-3.72) also reflected neutral to mildly positive attitudes. FC scored lowest (mean = 2.79-3.07), highlighting gaps in infrastructure. BI was moderate (mean = 2.98-3.27), suggesting cautious interest in adoption. SI (mean = 3.08-3.62) and HT (mean = 3.20-3.28) further supported AI acceptance. These findings underscore the importance of enhancing systemic support to translate favourable attitudes into practical adoption of AI in dental care (Table 2).

**Table 2:** UTAUT2 questionnaire mean scores.

Construct	Mean	±SD
**Performance expectancy**		
PE1. Using AI-powered diagnosis will improve the accuracy of my dental treatment	3.41	0.92
PE2. AI-assisted diagnosis will enhance my overall dental care experience	3.58	0.84
PE3. I believe that AI technology will help my dentist make better decisions	3.73	0.82
**Effort expectancy (ease of use)**		
EE1. I find it easy to understand how AI-powered diagnosis works	3.51	0.95
EE3. I will feel comfortable interacting with AI systems during my dental appointments	3.43	0.97
**Social influence**		
SI1. My family and friends believe we should use AI-powered diagnosis for dental care	3.08	0.97
SI2. Healthcare professionals should encourage the use of AI technologies in dental treatments	3.62	0.93
SI3. There is a general belief among patients that AI can improve dental diagnosis	3.33	0.92
**Facilitating conditions**		
FC1. I have access to the necessary technology to utilize AI-powered diagnosis in my dental visits	3.07	1.11
FC2. My dentist provides adequate support for understanding and using AI technologies	2.88	1.04
FC3. The dental clinic has the resources needed to implement AI-assisted diagnosis effectively	2.79	1.20
**Hedonic motivation**		
HM1. I enjoy using new technologies, including AI, in my healthcare	3.33	0.97
HM2. Interacting with AI systems during my dental visits will be fun for me	3.51	0.96
HM3. I find the use of AI in healthcare to be an exciting experience	3.72	0.86
**Habit**		
HT1. I regularly use technology for my healthcare needs, including dental care	3.20	1.00
HT2. Using technology, such as AI, has become a routine part of my healthcare experience	3.28	1.01
**Behavioural intention (BI)**		
BI1. I intend to use AI-powered dental diagnosis in the future	3.35	0.93
BI2. I would rely on AI-powered dental diagnosis, even when performed with less-experienced users.	3.27	0.99
BI3. I would prefer a dental clinic that uses AI diagnosis over one that relies only on traditional methods	2.98	1.07

SD, standard deviation.

The CFA indicated acceptable psychometric properties for most constructs within the proposed UTAUT2 model (Table 3). PE demonstrated excellent reliability (CR = 0.846, AVE = 0.648), with all factor loadings exceeding 0.70. HM also exhibited strong reliability (CR = 0.832, AVE = 0.623). SI (CR = 0.769) and FC (CR = 0.756) met acceptable reliability thresholds, although individual items FC2 (loading = 0.56) and SI3 (loading = 0.62) showed relatively lower factor loadings. In contrast, EE (CR = 0.608) and HT (CR = 0.547) fell below conventional standards, while BI demonstrated marginal reliability (CR = 0.651). Path analysis showed acceptable model fit indices, with *χ*² = 506.1, df = 114, *χ*²/df = 4.4, GFI = 0.900, CFI = 0.906, TLI = 0.907, and RMSEA = 0.083, indicating acceptable structural validity for SEM analysis.

**Table 3:** CFA Convergent validity and construct reliability after the deletion of measurement items.

Construct	Factor loadings	*R*^2^	CR	AVE
**Performance expectancy**			0.846	0.648
PE1	0.83	0.69		
PE2	0.83	0.69		
PE3	0.74	0.55		
**Effort expectancy**			0.608	0.439
EE1	0.59	0.35		
EE3	0.72	0.52		
**Social influence**			0.769	0.529
SI1	0.74	0.38		
SI2	0.80	0.64		
SI3	0.62	0.55		
**Facilitating conditions**			0.756	0.514
FC1	0.75	0.31		
FC2	0.56	0.64		
FC3	0.80	0.57		
**Hedonic motivation**			0.832	0.623
HM1	0.77	0.54		
HM2	0.85	0.72		
HM3	0.73	0.60		
**Habit**			0.547	0.382
HT1	0.51	0.26		
HT2	0.70	0.50		
**Behavioural intention**				
BI2	0.70	0.50	0.651	0.482
BI3	0.68	0.46		

AVE, average variance extracted; CR, composite reliability; *R*^2^, squared multiple correlation.

**Fig. 2:**
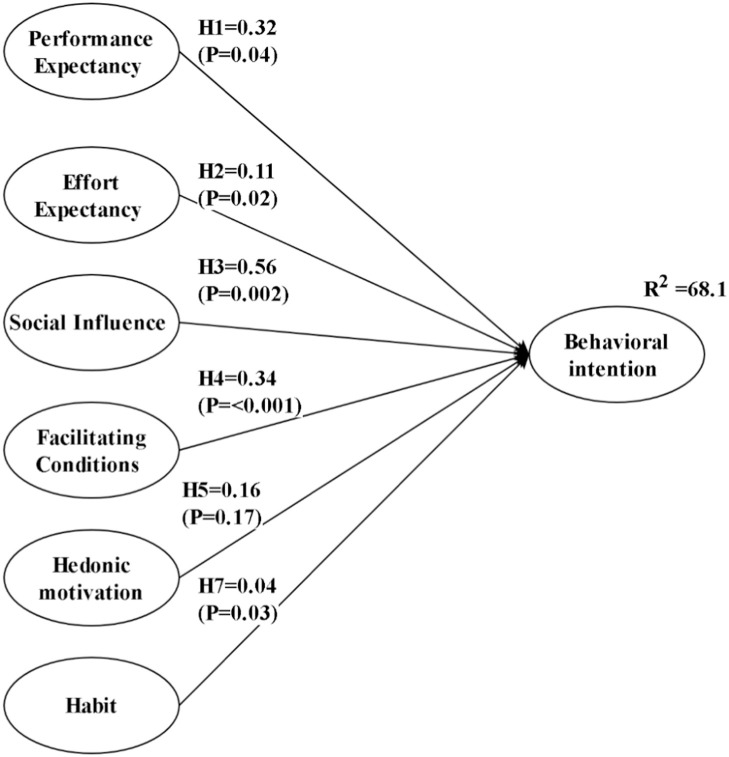
AMOS path analysis results from this study.

The path analysis shows that SI (*β* = 0.56, *P* = .002) and FC (*β* = 0.34, *P* < .001) strongly predict BI, which has an *R*² of 0.681, indicating that these factors explain 68.1% of the variance in BI. PE (*β* = 0.32, *P* = .04) and EE (*β* = 0.11, *P* = .02) have smaller but statistically significant effects. HM (*β* = 0.16, *P* = .17) was not significant, while HT (*β* = 0.04, *P* = .03) had minimal impact. Overall, the model demonstrates strong explanatory power for BI (Figure 2).

**Fig. 3:**
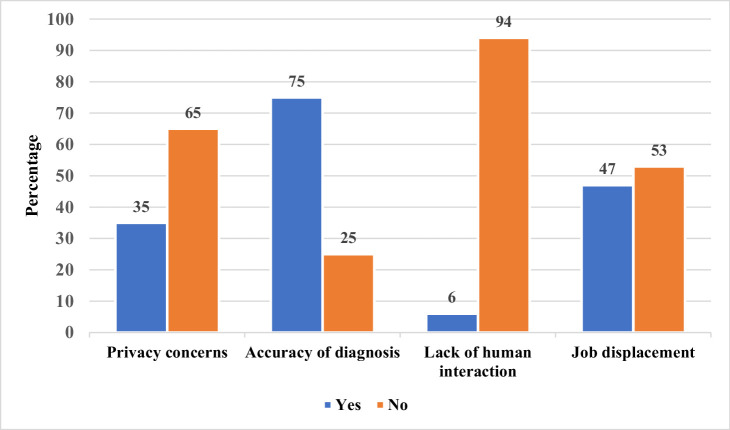
Distribution of concerns about AI use in dental diagnosis.

Most participants (75%) expressed concerns about the accuracy of AI dental diagnosis. 35% reported privacy concerns, while only 6% were concerned about the lack of human interaction when using AI systems (Figure 3).

Table 4 shows that all research hypotheses (H1-H6) were supported, confirming positive impacts on AI-powered diagnosis adoption.

**Table 4:** Results of the research hypotheses.

Research hypothesis	Result
H1. The impact of PE on BI to adopt AI-powered diagnosis will be positive.	Support
H2. The impact of EE on BI to adopt AI-powered diagnosis will be positive.	Support
H3. The impact of SI on BI to adopt AI-powered diagnosis will be positive.	Support
H4. The impact of FC on BI in adopting AI-powered diagnosis will be positive.	Support
H5. The impact of HM on BI to adopt AI-powered diagnosis will be positive.	Nonsupport
H6. The impact of HT on BI of AI-powered diagnosis will be positive.	Support

Females scored higher on HT (*P* = .027). Participants with prior AI experience reported significantly higher SI (*P* = .002) and HT (*P* = .003). Non-Saudis reported greater HM (*P* = .001). No significant gender-, experience-, or nationality-based differences were observed for PE, EE, or FC (Supplementary Table 2). Age significantly influenced FC (*P* = .042), while education level affected both PE (*P* = .009) and FC (*P* < .001). Other age- and education-related comparisons were nonsignificant (*P* > .05). These results suggest that FC is sensitive to demographic factors, while PE varies primarily with education. No other UTAUT2 constructs showed significant demographic differences (Supplementary Table 3).

## Discussion

The acceptance and practical application of AI in healthcare depend on multiple factors. Our study sample consisted largely of young, educated men from Saudi Arabia. This demographic profile suggests a high potential for AI adoption in dental care, underscoring the importance of understanding acceptance factors as described in the UTAUT2 model. Notably, 47% of respondents were aged 18 to 30, a group likely to be technologically adept and receptive to innovative AI applications in healthcare.^18^ The respondents’ high educational levels suggest a greater ability to understand complex technologies, aligning with general findings that educational background can influence technology acceptance.^19^ The majority of participants (57%) reported prior experience with AI technologies, indicating a familiarity that could facilitate smoother adoption in dental contexts.^20^^,^^21^

This study identifies key predictors of BI for using AI in dental diagnosis, based on the constructs of the UTAUT2 model. Both the mean scores and SEM results highlighted PE as a significant predictor of BI to adopt AI-powered diagnosis. This finding aligns with previous research emphasizing the role of perceived efficacy in healthcare technology adoption.^22^ Dashti et al’s^23^ systematic review also showed that PE significantly influences AI acceptance among dental students and practitioners. A strong validation also comes from a systematic review and meta-analysis of AI algorithms for gender detection using orthopantomograms, which demonstrated high diagnostic accuracy across multiple studies, reinforcing confidence in AI’s clinical reliability.^24^ PE reflects expectations of improvements in diagnostic accuracy, clinical decision-making, and overall user experience, consistent with evidence from systematic reviews showcasing AI’s transformative potential in healthcare settings.^25^^,^^26^ As clinicians increasingly recognize AI’s potential to enhance patient outcomes, their willingness to adopt AI-driven solutions is expected to grow. Recent developments in dental AI research emphasize patient satisfaction. Studies demonstrate the accuracy of AI-powered diagnosis using radiographs^24^ and highlight evolving perceptions among dental professionals and students.^23^ Comprehensive reviews^27^^,^^28^ emphasize AI’s transformative potential and contextual challenges, aligning with our findings on intention and trust in dental diagnosis.

While EE showed a positive correlation with BI (*β* = 0.11, *P* = .02), its impact was less substantial than that of PE. This finding is consistent with earlier studies,^22^ which suggest that although ease of use is important for adoption, perceived performance plays a more decisive role. The average EE scores indicate generally positive perceptions regarding the ease of understanding and interacting with AI systems. Simplifying user interfaces and providing comprehensive training could leverage this construct to enhance acceptance rates.^25^ Similarly, limited knowledge, especially about advanced AI topics like deep learning, has been reported as a major barrier despite an overall positive attitude.^23^ Samaranayake et al^27^ also emphasized the need for well-structured AI training in both undergraduate and continuing dental education, which aligns with our findings on educational background influences PE and EE.

Family and professional recommendations, as components of SI, strongly predicted technology acceptance, showing a positive correlation with BI.^29^ Findings revealed a strong participant belief in the role of healthcare professionals in advocating for AI in dentistry, aligning with studies demonstrating the impact of peer influence and social conformity on technology adoption.^30^ Considering this, social dynamics could be leveraged in outreach programs to increase healthcare providers’ acceptance of AI tools.

FC emerged as another important determinant of BI (*β* = 0.34, *P* < .001), although it had notably lower mean scores compared to other constructs. This finding supports the view that practical factors such as resource availability, technology access, and organizational support are critical for the successful implementation of AI technologies.^31^ The lower mean scores suggest perceived inadequacies in available resources, highlighting the need for a well-equipped information technology infrastructure to support AI applications in dental clinics. Enhancing clinic resources, providing technology training, and seamlessly integrating AI systems into existing workflows may improve user experience and reduce barriers to implementation. Infrastructure readiness, training, and access to technical support were similarly emphasized as crucial factors for successful AI integration.^23^ Tuygunov et al highlighted similar global barriers, particularly unequal access to digital infrastructure, underfunded health systems, and ethical ambiguity, suggesting that FC are not only technical but also systemic and geopolitical. Additionally, their report under the International Dental Federation (FDI) stresses the importance of data protection, transparency, and clinical accountability, factors that may directly influence users’ performance and effort expectancies.^28^

HM results suggest that the enjoyment and excitement associated with using AI contribute to its acceptance.^32^ While HM showed a positive trend in mean scores, it was not a significant predictor in the SEM model, indicating a limited influence on BI. Mean scores across constructs indicated neutral to mildly positive perceptions, suggesting cautious optimism toward AI use in dental diagnosis rather than strong endorsement.

It is interesting that HT minimally affects BI, indicating that habitual technology use does not automatically generate positive feelings toward AI in healthcare.^33^ Regular technology use does not automatically translate into adoption of AI diagnostic tools, suggesting a disconnect that warrants further research. This contrasts with previous findings where habitual technology users, particularly clinicians, were somewhat more open to AI, indicating experience-based trust.^23^

Gender, prior AI use, and nationality showed nuanced differences in independent-samples *t* tests, suggesting the need for tailored strategies to address resistance within specific demographics.^34^ For instance, women had higher HT scores, indicating greater habitual engagement, while individuals with prior AI experience showed increased awareness of SI.

Significant variations in both FC and PE across educational backgrounds highlight the critical role of education in shaping attitudes toward AI adoption.^35^ To address this gap, tailored educational interventions targeting underrepresented or less-educated groups are essential. Such initiatives can help demystify AI, build confidence in its use, and ensure more equitable adoption across all segments of the healthcare workforce. This supports the recommendation to embed AI literacy in dental curricula and continuing professional education, especially since many students report uncertainty despite enthusiasm.^23^

This study has several limitations. Its cross-sectional design limits causal inference. Self-reported data may introduce common method bias and social desirability effects. The online survey distribution may have excluded individuals with limited digital access, affecting generalizability. Future studies should consider longitudinal designs, objective measures, and more inclusive sampling to enhance validity and applicability. Additionally, the sample was skewed toward younger, educated individuals familiar with AI, limiting generalizability. Future research should include older adults and lower socioeconomic groups, whose perceptions may differ, to provide a more comprehensive understanding of AI acceptance in dental diagnosis.

## Conclusion

Acceptance of AI-powered dental diagnosis is influenced by multiple factors, as demonstrated by strong findings from this research. The crucial roles of PE, SI, and FC in shaping BI are particularly highlighted. Realizing AI’s potential in healthcare requires prioritizing systematic training, infrastructure development, and effective communication, all aligned with key adoption factors. These findings emphasize the need for healthcare providers, educators, and policymakers to work collaboratively to successfully integrate AI into routine healthcare practice.

## Author contributions

S.N. and N.G.T.: conceptualization, data curation, formal analysis, investigation, methodology, validation, visualization, writing original draft, review, and editing. S.V.: conceptualization, data curation, methodology, resources, software, supervision, visualization, writing review, and editing. M.A. and A.A.A.K.: conceptualization, data curation, formal analysis, investigation, methodology, resources, supervision, validation, writing review, and editing. Y.F.A. and Z.M.A.: conceptualization, data curation, funding acquisition, methodology, project administration, resources, supervision, writing review, and editing. T.M.A. and A.C.: conceptualization, data curation, formal analysis, methodology, resources, supervision, writing review, and editing. All authors have materially participated in the research and/or article preparation, provided final approval of the submitted version, and agreed to be accountable for all aspects of the work, ensuring its integrity and accuracy.

## Ethics statement

The study received approval from King Saud University’s Institutional Review Board (IRB No: E-24-9321) and was conducted in accordance with committee standards.

## Data availability

The data supporting the findings of this study are available from the corresponding author upon reasonable request.

## Conflict of interest

The authors declare that they have no known competing financial interests or personal relationships that could have appeared to influence the work reported in this article.

## Appendix Supplementary materials

Image, application 1

## Footnotes

Supplementary material associated with this article can be found in the online version at doi:10.1016/j.identj.2025.103893.
