# Supplementary material for: Patients’ Acceptance and Intentions on Using Artificial Intelligence in Dental Diagnosis: Insights From Unified Theory of Acceptance and Use of Technology 2 Model
Source: Int Dent J. 2025 Sep 6;75(6):103893. doi: 10.1016/j.identj.2025.103893 (PMC12452871; doi:10.1016/j.identj.2025.103893)
Supplement: Supplementary file 1 [file mmc1.docx]

| Hypothesis | References |
| --- | --- |
| H1 | Consumer Acceptance and Use of Information Technology: Extending the Unified Theory of Acceptance and Use of Technology. |
|  | Mobile Government Adoption Model Based on Combining GAM and UTAUT to Explain Factors According to Adoption of Mobile Government Services |
|  | Adoption of Clinical Decision Support Systems in a Developing Country: Antecedents and Outcomes of Physician’s Threat to Professional Autonomy |
| H2 | Consumer Acceptance and Use of Information Technology: Extending the Unified Theory of Acceptance and Use of Technology. |
|  | Mobile Government Adoption Model Based on Combining GAM and UTAUT to Explain Factors According to Adoption of Mobile Government Services |
| H3 | Consumer Acceptance and Use of Information Technology: Extending the Unified Theory of Acceptance and Use of Technology. |
|  | Adoption of Clinical Decision Support Systems in a Developing Country: Antecedents and Outcomes of Physician’s Threat to Professional Autonomy |
| H4 | Consumer Acceptance and Use of Information Technology: Extending the Unified Theory of Acceptance and Use of Technology |
| H5 | Consumer Acceptance and Use of Information Technology: Extending the Unified Theory of Acceptance and Use of Technology. |
|  | Patient Acceptance of AI in Dentistry: The Role of Perceived Enjoyment and Trust (Link) – Explores how enjoyment (HM) and habitual use (HT) drive patient adoption. |
| H6 | Consumer Acceptance and Use of Information Technology: Extending the Unified Theory of Acceptance and Use of Technology. |
|  | Patient Acceptance of AI in Dentistry: The Role of Perceived Enjoyment and Trust (Link) – Explores how enjoyment (HM) and habitual use (HT) drive patient adoption. |

**Table 1. References for the construction of the conceptual model**

**Table 2- Independent Sample T-test of gender, Prior AI use, nationality**

|  | Gender | |  | Prior AI use | |  | Nationality | |  |
| --- | --- | --- | --- | --- | --- | --- | --- | --- | --- |
|  | Male | Female | *P* value | Yes | No | *P* value | Saudi | Non- Saudi | *P* value |
| PE | 3.59 | 3.55 | 0.381 | 3.67 | 3.50 | 0.510 | 3.57 | 3.62 | 0.166 |
| EE | 3.39 | 3.58 | 0.572 | 3.58 | 3.39 | 0.801 | 3.44 | 3.66 | 0.211 |
| SI | 3.24 | 3.48 | 0.625 | 3.48 | 3.24 | 0.002* | 3.27 | 3.78 | 0.818 |
| FC | 2.84 | 3.00 | 0.128 | 2.98 | 2.86 | 0.598 | 2.87 | 3.16 | 0.780 |
| HM | 3.45 | 3.61 | 0.640 | 3.60 | 3.46 | 0.175 | 3.48 | 3.74 | 0.001* |
| HT | 3.15 | 3.36 | 0.027* | 3.40 | 3.12 | 0.003* | 3.23 | 3.31 | 0.257 |

PE (Performance Expectancy), EE (Effort Expectancy), SI (Social Influence), FC (Facilitating Conditions), HM (Hedonic Motivation), HT (Habit).

**Table 3- One-way ANOVA for other analysis**

| Variables | Facet | F-test | P-Value |
| --- | --- | --- | --- |
| Age | PE | 1.293 | 0.092 |
|  | EE | 1.304 | 0.086 |
|  | SI | 1.163 | 0.215 |
|  | FC | 1.369 | 0.042* |
|  | HM | 1.315 | 0.079 |
|  | HT | 1.342 | 0.064 |
| Education level | PE | 3.892 | 0.009* |
|  | EE | 1.294 | 0.276 |
|  | SI | 1.734 | 0.159 |
|  | FC | 7.362 | 0.000* |
|  | HM | 2.270 | 0.080 |
|  | HT | 1.481 | 0.219 |
